# Exciton-Defect Interaction and Optical Properties from a First-Principles T‑Matrix Approach

**DOI:** 10.1021/acs.nanolett.5c04479

**Published:** 2026-01-13

**Authors:** Yang-hao Chan, Jonah B. Haber, Mit H. Naik, Diana Y. Qiu, Felipe H. da Jornada

**Affiliations:** † Institute of Atomic and Molecular Sciences, 38017 Academia Sinica, Taipei 10617, Taiwan; ‡ Department of Materials Science and Engineering, 6429 Stanford University, Stanford, California 94305, United States; § Department of Physics, University of Texas at Austin, Austin, Texas 78712, United States; ∥ Department of Materials Science, 5755 Yale University, New Haven, Connecticut 06520, United States

**Keywords:** exciton-defect interaction, absorption, photoluminescence, T-matrix, first principles

## Abstract

Understanding exciton-defect interactions is critical for optimizing optoelectronic and quantum information applications in many materials. However, *ab initio* simulations of material properties with defects are often limited to high defect density. Here, we study effects of exciton-defect interactions on optical absorption and photoluminescence spectra in monolayer MoS_2_ using a first-principles T-matrix approach. We demonstrate that exciton-defect bound states can be captured by the disorder-averaged Green’s function with the T-matrix approximation and further analyze their optical properties. Our approach yields photoluminescence spectra in good agreement with experiments and provides a new, computationally efficient framework for simulating optical properties of disordered 2D materials from first-principles.

## Graphical abstract

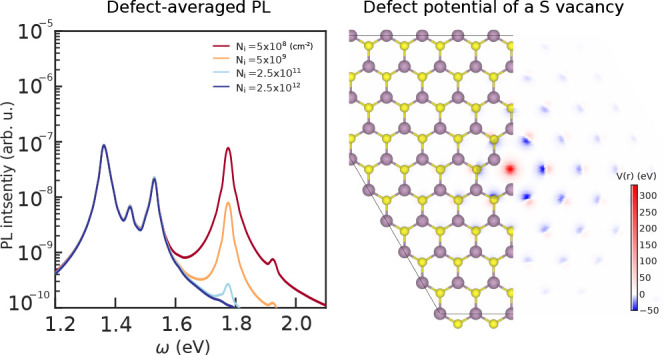

Optical properties of materials can be strongly affected by the presence of defects through changes of energy levels and introduction of new in-gap states.^1–7^ In quasi-two-dimensional (2D) systems, strongly bound excitons (correlated electron–hole pairs) dominate the low-energy optical spectra. A thorough understanding of exciton-defect interactions is critical to the fundamental understanding of exciton character and dynamics in disordered systems^8–10^ and is essential for optoelectronic devices and quantum information applications.^11–16^

State-of-the-art calculations of optical properties with defects, utilizing a supercell approach, have been performed at the GW ( where G and W denote the one-particle Green function and the screened Coulomb interaction, respectively) plus Bethe-Salpeter equation (BSE) level, where both quasi-particle energy renormalization and electron–hole interactions are taken into account.^7,17,18^ However, due to the large computational cost associated with calculating excited states in large supercells, only systems with high defect density, where defect–defect interactions and artificial periodicity might obscure single-defect properties, have been studied. A thorough investigation of the defect density dependence of the optical spectrum, especially in low-density experimentally relevant regimes, has not yet been conducted.

One way of accessing the dilute defect limit is to treat the defect potential as a perturbation acting on the excitonic states of the pristine system: in this picture, the exciton propagates through the host material and is scattered by the localized potential introduced by the defect. The lowest order treatment of this interaction—the Born approximation—accounts for single scattering events but cannot capture defect-bound states, where it is necessary to coherently resum scattering events to infinite order. This infinite resummation, encapsulated by the T-matrix formalism, allows poles to build in the exciton Green’s function, corresponding to defect-bound states. The T-matrix formalism has recently been applied for electron-defect problems from first-principles, where electron-defect scattering rates and defect-bound states are analyzed for several materials.^19^

In this paper, we develop an efficient *ab initio* T-matrix approach to study exciton-defect interactions applicable to a wide range of defect densities. We find that, in addition to the A and B peaks of MoS_2_, a weak absorption peak appears when defects are included, which can be identified as a defect-bound state. We benchmark two common methods to incorporate disorder-averaged self-energies, and show that, unlike the T-matrix, the Born approximation is incapable of qualitatively capturing defect-bound excitons. We compute the photoluminescence (PL) spectra including defect scattering from first-principles and observe clear signals from defect-bound states that completely dominate the PL spectrum at low temperatures, despite their lower oscillator strengths, in good agreement with available experiments. We anticipate that this framework will open new avenues for investigation into exciton-defect interaction, enabling systematic studies across a range of defect densities at minimal computational cost—ultimately laying the groundwork for understanding even more complex processes, e.g. inelastic exciton-defect scattering. We note that the T-matrix approach is however appropriate for understanding the evolution of spectra properties in the dilute defect limit^19,20^ and may not be appropriate for studying specific isolated defect configurations, as explored in recent work.^21^

We start by writing a Hamiltonian, defined in the primitive unit cell of a material, describing a set of excitons that can scatter with defects,

1 $$H=\underset{S,Q}{\sum }E_{SQ}a_{SQ}^{†}a_{SQ}+\underset{SS^{\prime }\mathrm{Qq}}{\sum }V_{SQ+q,S\prime Q}a_{SQ+q}^{†}a_{S\prime Q}$$

where *a*_*S* **Q**_ (*a*_*S* **Q**_^†^) are exciton annihilation (creation) operators for an exciton state *S* with finite center of mass momentum (COM) **Q** and *V*_*S* **Q**+**q**,*S*′**Q**_ describes the scattering amplitude between exciton state (*S*′, **Q**) and (*S*, **Q** + **q**). In this work, we consider the sulfur-vacancy (S-vacancy) defect, which is commonly observed in MoS_2_.^7,9,16^ The defect potential is extracted from density-functional theory (DFT) calculations with a relatively small supercell and requires no explicit knowledge of Kohn–Sham states in large supercells corresponding to the defect densities of interest.^19,22–25^ The single S-vacancy defect potential is shown in Figure 1(a) for a 9 × 9 × 1 supercell and the resulting exciton density of states of eq 1 is shown in Figure 1(b). The exciton-defect scattering matrix element is calculated as

2 $$V_{SQ+q,S\prime Q}=\underset{k}{\sum }\left[\underset{vcc^{\prime }}{\sum }A_{ck+Q+q,vk}^{S*}A_{c\prime k+Q,vk}^{S\prime }V_{cc\prime }\left(k+Q,q\right)-\underset{cvv^{\prime }}{\sum }A_{ck+Q,vk-q}^{S*}A_{ck+Q,v\prime k}^{S\prime }V_{v\prime v}\left(k-q,q\right)\right]$$

where *A*_*cv* **k**_^*S*^ is the exciton envelope function obtained by solving the BSE^26–28^ and *V*_*nm*_(**k**, **q**) is the electron-defect matrix element between electron states (*m*, **k**) and (*n*, **k** + **q**). The first (second) term in eq 2 describes electrons (holes) scattering off defects. The details of the calculations are given in the Supporting Information (SI).

**Figure 1:**
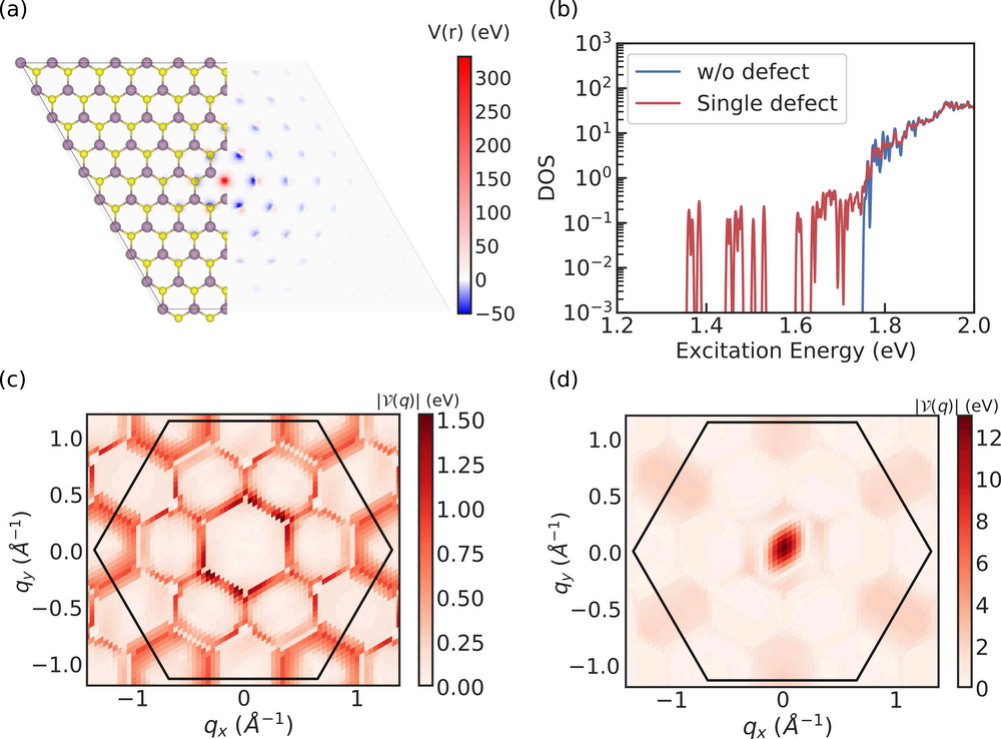
(a) Intensity map of a single S-vacancy defect potential in a 9 × 9 supercell overlaid with the atomic model, where purple balls are Mo atoms and yellow balls are S atoms. (b) Density of exciton states without (blue) and with (red) the single S-vacancy defect in the supercell. Absolute value of defect scattering matrix elements between A exciton and excitons (c) in the first and (d) the third exciton band in the Brillouin zone.

We show in Figure 1(c) and (d) the exciton-defect matrix elements between the A exciton, with **Q = 0**, and other excitons with COM **q** in the first and third exciton bands, respectively. We observe that the momentum distribution of the matrix elements slightly breaks the 3-fold rotation symmetry, which is due to structural relaxation in our defect simulations. The vanishing matrix elements near **q** = 0 in Figure 1(c) and the large amplitude in (d) can be understood from spin quantum number conservation. Since the S-vacancy does not induce spin-flip processes and spin is almost a good quantum number for low energy excitons in monolayer MoS_2_,^30,31^ the A exciton predominantly scatters into parallel-spin states.

Up to this point, we have focused on the single defect-per-supercell problem described by eq 1, which can be solved by exact diagonalization. However, one would like to access various—possibly dilute—defect concentrations and avoid explicitly diagonalizing eq 1, since one would need to solve the BSE for additional COM **Q** and consistently utilize a finer **k**-point grid for different defect densities, which quickly becomes the bottleneck of the approach, as opposed to the exact diagonalization of eq 1 itself. We achieve the practical solution of the exciton-defect problem within our Green’s function approach through a disorder-averaging procedure.^19,20^ Importantly, such a defect-averaging process recovers the translational symmetry of the primitive unit cell of the material—broken by an isolated defect or array of defects in a supercell—and leads to a self-energy diagonal in the exciton’s COM crystal momentum. In the following, we adopt Born and T-matrix self-energy approximations and compare their self-energy, Green’s function, and the absorption spectrum. The self-energy from Born approximation describes scattering processes shown in the inset of Figure 2(a). In contrast, the quasiparticle can scatter off defects multiple times in the T-matrix approximation as illustrated in the inset of Figure 2(b). While the Born self-energy is the lowest order nontrivial one, it has been shown that the T-matrix approximation becomes exact in the low defect density limit.^20^

**Figure 2:**
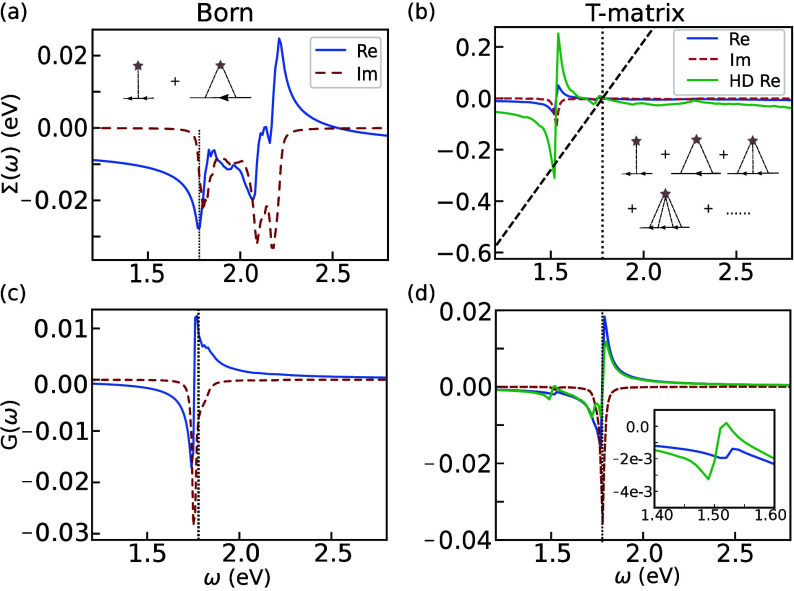
Retarded (a) Born and (b) T-matrix self-energy of A exciton calculated with a defect density of 5 × 10^11^ cm^–2^. (c) and (d) show the corresponding Green’s functions. Blue solid lines (red dashed lines) are the real (imaginary) part. Black dotted lines mark the bare A exciton energy. In (b) and (d), the real part of the self-energy and the Green’s function with a defect density of 2.5 × 10^12^ cm^–2^ are shown as green solid lines, respectively. The intersection between the dashed diagonal line and the real part of the self-energy gives the bound state solution. Insets in (a) and (b) show the self-energy diagrams in each approximation. The inset in (d) is a zoom-in view around 1.5 eV.

The Born self-energy is written as

3 $$\Sigma_{SS\prime Q}^{B}\left(\omega \right)=N_{i}\left[V_{SQ,S\prime Q}+\underset{S^{\prime \prime }Q^{\prime }}{\sum }V_{SQ,S\prime \prime Q\prime }G_{S\prime \prime Q\prime }^{0}\left(\omega \right)V_{S\prime \prime Q\prime ,S\prime Q}\right]$$

where *N*_*i*_ is the number of defects, and *G*_*S* **Q**_^0^ = 1/(ω – *E*_*S* **Q**_ + *iη*) is the retarded bare exciton Green’s function with η = 10 meV in our calculations. For the T-matrix self-energy, we have Σ_**Q**_^*T*^(ω) = *N*_*i*_ *T*_**QQ**_(ω) with^19,20^

4 $$T_{SQS\prime Q\prime }\left(\omega \right)=V_{SQS\prime Q\prime }+\underset{S^{\prime \prime }Q^{\prime \prime }}{\sum }\left[V_{SQS\prime \prime Q\prime \prime }\times G_{S\prime \prime Q\prime \prime }^{0}\left(\omega \right)T_{S\prime \prime Q\prime \prime S\prime Q\prime }\left(\omega \right)\right]$$

The full Green’s function is solved by inverting Dyson’s equation, *G*(ω)^−1^ = *G* ^0^(ω)^−1^ – Σ­(ω) for each frequency ω from which we obtain the exciton spectral function including defect scattering.

In Figure 2, we show the self-energy and the Green’s function in the two approximations for the A exciton with a defect density of 5 × 10^11^ cm^–2^. Notably, the Born self-energy is featureless below the bare exciton energy, defined as that in the material without defects, while the T-matrix self-energy acquires a lower-energy pole at 1.53 eV, which can be assigned as the defect-bound state and will be denoted as “Bd1” in the following (see Supporting Information). As a result, a corresponding secondary peak appears in the T-matrix Green’s function. This striking difference between the Born and T-matrix self-energy is well-documented: because the Born approximation accounts for a finite (two) scattering events between excitons and defects, it cannot capture bound states. At most, it describes a renormalization of the exciton energies due to the change of the average potential they experience. In contrast, the T-matrix allows for an infinite number of scatering events between excitons and defects, allowing for a bound state to emerge.

As the defect density increases, the Born and T-matrix approximations also display contrasting behaviors. In the Born approximation, the renormalization of the exciton energy increases with the defect density, without the appearance of any lower-energy peak associated with a defect-bound exciton. In contrast, for the T-matrix, as the defect density increases, more spectral weight gets transferred to the lower-energy secondary peak, with little renormalization of the bare exciton energies. The T-matrix approximation hence correctly captures the physics that there are more defect-bound excitons in highly disordered samples, while the Born approximation is qualitatively incapable of describing excitons in defected materials. The defect density dependence of both self-energies and Green functions of A exciton is given in SI.

The absorption spectrum including the exciton-defect interactions can be calculated from the retarded Green’s function, ϵ2(ω)=−e2ϵ0VtotIm⁡∑SS′ΩS′*GS′SQ=0R(ω)ΩS where *V*_*tot*_ is the crystal volume, Ω_*S*_ = ∑_*cv* **k**_ *A*_*cv* **k**_^S*^ *d*_*cv* **k**_ are exciton dipole matrix elements, and *d*_*vc* **k**_ are electron dipole matrix elements.

In Figure 3(a) and (b) we show the spectra from T-matrix and Born approximations, respectively, at two defect densities and for the pristine case. As expected from the Green’s function calculations, we observe that A and B peaks shift to lower energy in Born approximation due to spurious exciton energy renomalizations with the average defect potential. In contrast, within the T-matrix approximation, their energy renomalizations are minimal. We instead observe the appearance of secondary peaks and shoulder structures, which can be identified as defect-bound states, and suppression of absorbance of A and B excitons. We also find that the Bd1 peak shifts to 1.5 eV at a defect density of 2.5 × 10^12^ cm^–2^, following the defect density dependence of the spectral function discussed earlier. To understand how defect-bound states acquire oscillator strength and their compositions of bare excitons, we write the full Green’s function as

5 $$G_{SS\prime }^{R}\left(\omega \right)=\underset{\lambda }{\sum }\frac{T_{\lambda S}\left(\omega \right)T_{\lambda S\prime }^{-1}\left(\omega \right)}{\hbar \omega -{Ẽ}_{\lambda }\left(\omega \right)-i{\mathrm{\Gamma ̃}}_{\lambda }\left(\omega \right)}$$

where *Ẽ*_λ_(ω) – *iΓ̃*_λ_(ω) and *T*_*λS*_(ω) are eigenvalues and eigenvectors, respectively, of the effective Hamiltonian *H*_*SS*′_(ω) = *E*_*S* **0**_δ_*SS*′_ + Σ_*SS*′_(ω). We obtain the same spectral function as by computing the full Green’s function *G*(ω) from diagonalizing *H*_*SS*′_(ω). In Figure 3(c), we plot AS(ω)=−1πIm⁡GSSR(ω) for selected exciton states at a defect density of 2.5 × 10^12^ cm^–2^. Compared with Figure 3(a), we find that the Bd1 peak has an origin of bare A exciton states—a fact that is also reflected in similar real-space distribution of the electron in the defect-bound exciton and A exciton shown in Figure 3(d) (see SI)—while the 1.75 eV peak has contributions from both A and B excitons. In addition to bright states, we find a lower-energy peak at 1.45 eV originating from dark, spin-unlike excitons (*D*_*A*_) in the pristine system, so they do not appear in the absorption spectrum. These states, and others extending up to ∼1.6 eV, can be connected to the low-energy eigenstate of eq 1. A general expression of *A*(ω) in terms of the eigenstates of eq 1 is given in SI, where a clear connection to bound states of the single defect problem can be made. For higher-energy excitons, we do not find associated defect-bound states, but their spectra extend a few hundred meV below the main peaks.

**Figure 3:**
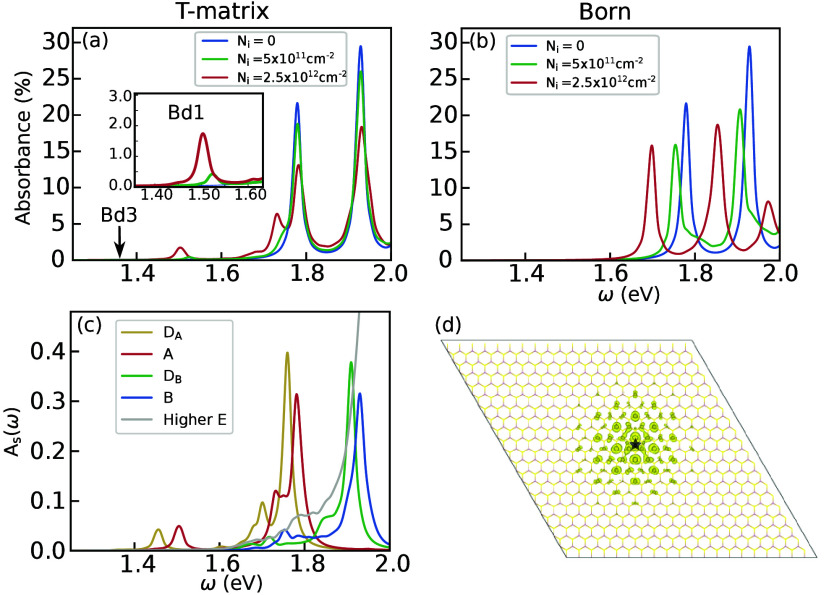
Computed optical absorbance for monolayer MoS_2_ within the (a) T-matrix and (b) Born approximations at several defect densities at 300 K. The inset in (a) shows a zoom-in view near the 1.5 eV. The arrow indicates the energy of the Bd3 defect-bound exciton. (c) Exciton-state decomposed spectral function *A*(ω) from T-matrix calculations for a defect density of 2.5 × 10^12^ cm^–2^, which shows the contributions from the bare exciton states. *D*_*A*_ and *D*_*B*_ refers to the lowest-energy dark states in the same series as A and B excitons, respectively. (d) Local electron density of states of the lowest energy peak in (a) for a fixed hole position marked by a star symbol.

The effect of defects on the PL spectrum has been carefully studied in several experiments^1,2,7,8,16^ but has only been investigated from first-principles in a few works.^17,18^ In contrast to the absorption, PL intensity is proportional to the lesser Green’s function^32,33^ as *I*_*PL*_(ω) ∝ ∑_*S*_ |Ω_*S*_|^2^ *G*_*SS* **0**_^<^(ω). In general, *G*_*SS* **Q**_^<^(ω) can be obtained from the Kubo-Martin-Schwinger relation, where *G* ^<^(ω) = *b*(ω)­(*G* ^*R*^(ω) – *G* ^*A*^(ω)) with *b*(ω) being the Bose distribution function. However, it is difficult to deal with the Bose function numerically when Im *G* ^*R*^ has a nontrivial structure, such as multiple sattelite peaks. Here, we calculate *G*_*SS*_^<^(ω) within a quasiparticle expansion^33–35^ which separates the quasiparticle part and the dynamical contribution as

6 $$G_{\mathrm{SS}Q}^{<}\left(\omega \right)=-2\pi \mathrm{ib}\left(\omega \right)\left[\delta \left(\omega -E_{SQ}\right)\left(1-R_{SQ}\right)+\frac{N_{i}}{{\left(\omega -E_{SQ}\right)}^{2}+\eta^{2}}\underset{\lambda }{\sum }|W_{SQ}^{\lambda }{|}^{2}\delta \left(\omega -E^{\lambda }\right)\right]$$

where *R*_*S* **Q**_ is the renormalization factor, *E* ^λ^ is the eigenenergy of eq 1, and *W*_*S* **Q**_^λ^ is the exciton-defect matrix elements projected to the eigenvector of eq 1 (see Supporting Information). The first term in eq 6 describes the renormalized quasiparticle peak with a reduced weight, while the second term is responsible for structures such as sattellites, obtained from dynamical effects. In this form of expressing *G* ^<^, the positions of the secondary peaks are solely determined by the energy of the discrete, defect-bound excitons from eq 1 that is solved for a fixed defect density. Therefore, we can approximately associate those peaks obtained at various defect densities with defect-bound states shown in Figure 1(b).

In Figure 4(a), we show the simulated PL intensity at 300 K for several defect densities, where the spectra are normalized by the total number of excitons. Besides the A, B, and Bd1 peaks at 1.78, 1.93, and 1.53 eV, we identified two additional defect-bound state emissions at 1.45 and 1.36 eV, denoted as “Bd2” and“Bd3”, respectively. We find that the Bd3 state consists of both A exciton and the lowest energy **Q** = 0 dark exciton, with the latter having about 4 times larger weight. Bd1 and Bd2 states are both derived from the A exciton. Notably, the detunings of the Bd1 and Bd2 peak with respect to the A peak are consistent with those observed in ref.,^16^ where emissions with detuning of 195 and 275 meV were reported. The lack of clear experimental evidence for the Bd3 peak could be related to its weak oscillator strength: It implies that its emission is only possible if the system reaches thermal equilibrium and there are no other decay and scattering mechanisms faster than the Bd3 radiative recombination time.

**Figure 4:**
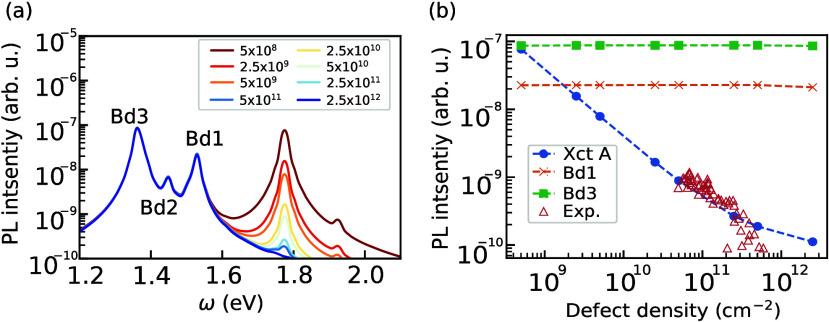
Simulated photoluminescence spectra at (a) different defect density at 300 K. Defect densities are in the unit of cm^–2^. (b) Defect density dependences of the PL intensity at A exciton (blue dots), Bd1 (orange crosses), and Bd3 (green squares) peaks. Experimental data in (b) is reproduced from ref 8 and scaled by an overall factor to align with the calculations. Copyright 2018, American Chemical Society.

At low defect density, the A peak intensity is comparable to the intensity of the defect states. With increasing defect density, A peak emission decreases rapidly, and the emission from the two bound states dominates the spectrum, since the PL intensity is directly proportional to exciton populations. The first term in eq 6 is only weakly dependent on defect densities, while the second term is proportional to it. Therefore, the A peak intensity must be inversely proportional to the defect density after normalization by the total number of excitons for each curve, as is indeed seen in Figure 4(b). Our results for monolayer MoS_2_ are in remarkable agreement with available experimental data for monolayer WS_2_.^8^ We attribute this agreement to similarities in the composition and electronic structure of these two closely related TMDs.

In conclusion, we develop a first-principles T-matrix approach for exciton-defect problems and apply it for S-vacancy in monolayer MoS_2_, where optical absorption and PL intensity spectra are simulated. We identified defect-bound states in both spectra and revealed their characters in terms of excitons in pristine MoS_2_. Compared against the Born approximation, we found a T-matrix approach is necessary to capture defect-bound states because the Born approximation merely shifts the exciton energy. The defect density dependence of the PL intensity and defect-bound state energy agrees reasonably well with experiment. Our approach can generally be applied to other materials and different types of defects. We anticipate that our approach can provide an understanding of exciton-defect couplings and the defect-bound exciton emission that complement the conventional supercell method limited to the high defect density.

## Supplementary Material



## Notes

Computational details of GW-BSE, exciton-defect effective Hamiltonian, expressions for optical absorption and photoluminescence spectra, temperature dependence of photoluminescence spetrum, and defect density dependence of exciton-defect self-energy and exciton Green function (PDF)
