# Supplementary material for: Exciton-Defect Interaction and Optical Properties from a First-Principles T‑Matrix Approach
Source: Nano Lett. 2026 Jan 13;26(3):961–6. doi: 10.1021/acs.nanolett.5c04479 (PMC12856894; doi:10.1021/acs.nanolett.5c04479)
Supplement: Supplementary file 1 [file nl5c04479_si_001.pdf]

Supporting Information of Exciton-defect interaction and optical properties from a first-principles T-matrix approach

Yang-hao Chan,[1,*] Jonah B. Haber,[2] Mit H. Naik,[3] Diana Y. Qiu,[4,†] and Felipe H. da Jornada[2,‡]

[1] *Institute of Atomic and Molecular Sciences, Academia Sinica, Taipei 10617, Taiwan*
[2] *Department of Materials Science and Engineering, Stanford University, Stanford, CA 94305, USA*
[3] *Department of Physics, University of Texas at Austin, Austin, TX, 78712, USA*
[4] *Department of Materials Science, Yale University, New Haven, CT 06520*

(Dated: November 24, 2025)

COMPUTATIONAL DETAILS

Electronic structure and density functional perturbation theory calculations are performed with the Quantum Espresso package [1]. Electron-defect matrix elements are computed with the Perturbo package [2–4]. The single S-vacancy defect potential is computed with a 9×9 supercell. We compute GW quasi-particle energy and solve for excitons with the BerkeleyGW package [5–7]. BSE are solved with 4 valence and 4 conduction bands on a 48×48 $\mathbf{k}$-grid. Finite COM excitons and exciton-defect coupling matrix elements are computed on a 48×48 $\mathbf{Q}$-grid [8]. The single defect problem in Eq. 1 is solved by including 20 exciton states at each COM. All other parameters can be found in our previous work [9].

ELECTRON-DEFECT HAMILTONIAN

In this section, we provide results for the electron-defect problem for monolayer MoS_2 with S-vacancy, which includes electron-defect matrix elements, bound-states and density of states (DOS) for a single-defect calculations, and the results from T-matrix approximations, which serve as a starting point to the exciton-defect problem.

The electron-defect interaction Hamiltonian for a single defect problem reads [3, 10, 11],

$$H_{e-i} = \sum_{ij} c_i^\dagger c_j g_{ij}. \tag{S1}$$

where the electron-defect scattering matrix g_{ij} between the state i and j is

$$g_{ij} = \langle i \left| \Delta V^i \right| j \rangle,$$

where $\Delta V^i = V^i - V^0$ is the potential difference of the system with defects and the pristine system. Because the defect potential $\Delta V(\mathbf{r})$ decays quickly as a function of the distance $\mathbf{r}$ with respect to the defect site, one can extract it from a relatively small supercell, and then evaluate arbitrary matrix elements of $\Delta V(r)$ between Kohn-Sham states. For instance, in our calculations, we extract the defect potential from an explicit 9×9 supercell calculation. Thereafter, we evaluate matrix elements g_{ij} between Kohn-Sham states of the pristine system on a $48\times48\times1$ grid, where the integral to compute g_{ij} is defined over the whole crystal spanned by an array of 48×48 primitive cells. Critically, because of its decay in real space, we don't need to explicitly perform a DFT calculation on a 48×48 supercell to obtain g_{ij}. In Fig. S1, we show the elements of intraband electron-defect coupling matrix, $g_{n\mathbf{k}+\mathbf{q},n\mathbf{k}}$ for the top of the valence band and the bottom of the conduction band at $\mathbf{k} = \mathbf{K}$ calculated with the Perturbo package [2–4]. The results are similar to those reported for WSe_2 in Ref. [10].

Next, we study a single-defect problem by diagonalizing Eq. S1 together with the non-interacting part of the Hamiltonian. The Hamiltonian is constructed with 4 conduction and valence states and a 48×48 $\mathbf{k}$-grid is used. In Fig. S2, we show the electron density of states together with that from DFT calculations. We can see that two in-gap states appear in our calculations although their energy deviates from the DFT results. To confirm that the in-gap states are indeed defect-bound states, we plot their charge distributions in Fig. S2 (b), where we can clearly see the charge localized around the S-vacancy. We note that in principle a fully converged calculation requires the inclusion of all the valence and conduction bands in the DFT calculations [10], which is, however, infeasible for the exciton-defect problem. Nevertheless, our calculations qualitatively reproduced the defect-bound states, which is essential for the defect-bound excitons.

We further calculate T-matrix selfenergy and the disorder-averaged Green's function for the electron-defect problem with formula similar to that of excitons given in the main text. In Fig. S3 the density of states computed from T-matrix approximations and that of the diagonalization are shown together with that of a pristine MoS_2. We find that the DOS computed with the T-matrix approximation agrees excellently with that of the diagonalization results, which indicates that the T-matrix calculations is indeed exact in the low density limit.

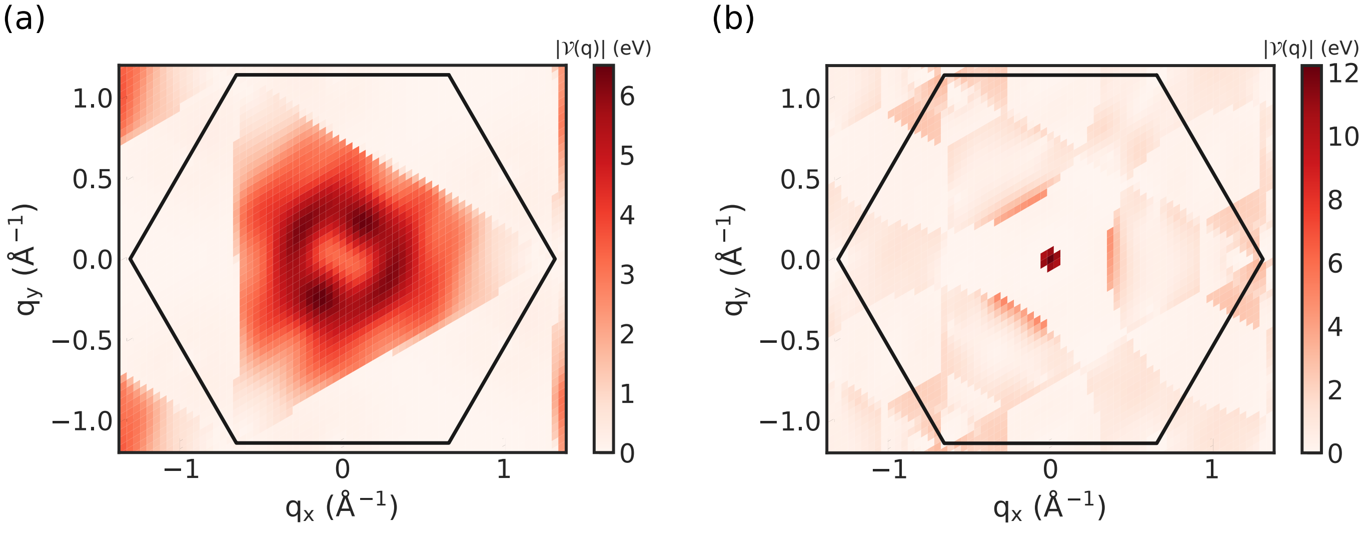

FIG. S1. Color maps of the absolute value of the intraband electron-defect matrix elements of the (a) top of the valence band and (b) bottom of the conduction band at **K** shown as a function of momentum transfer **q**.

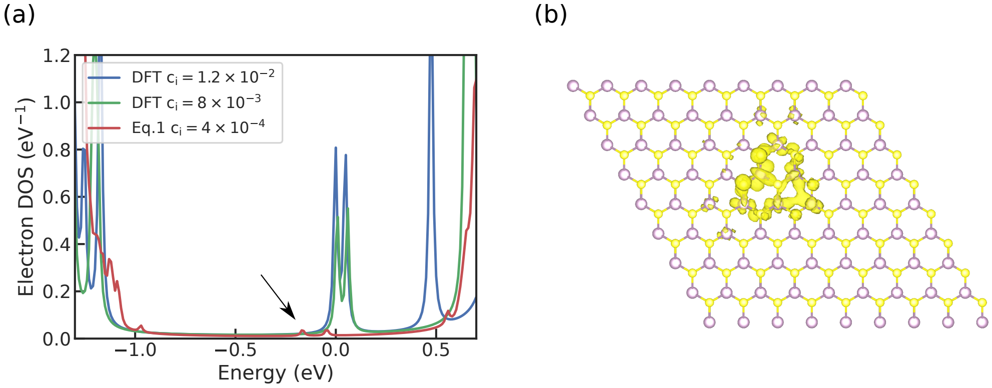

FIG. S2. (a) Electronic density of states of monolayer MoS_2 calculated from DFT at a defect density of $c_i = 1.2 \times 10^{-2}$ (blue) per unit cell, $8{\times}10^{-3}$ (green), and computed from diagonalizing the single-defect Hamiltonian at $c_i = 4 \times 10^{-4}$ (red) per unit cell. The arrow indicates the in-gap state which we show the charge distribution in (b).

EXCITON-DEFECT HAMILTONIAN

Starting from Eq. S1, we derive the effective exciton-defect interaction following Ref. [12]. The perturbed BSE Hamiltonian in the unperturbed exciton basis is written as

$$\tilde{H}_{mn} = \langle S_m \left| \tilde{H} \right| S_n \rangle = \sum_{vc,v'c'} A_{vc}^{S_m *} A_{v'c'}^{S_n} \times \left[\sum_{\tilde{v}\tilde{c},\tilde{v}'\tilde{c}'} \langle vc|\tilde{v}\tilde{c}\rangle \langle \tilde{v}\tilde{c} \left| \tilde{H} \right| \tilde{v}'\tilde{c}'\rangle \langle \tilde{v}'\tilde{c}'|v'c'\rangle \right],$$

where $\tilde{H}$ and $\tilde{v}\tilde{c}$ are the Hamiltonian and electron-hole wavefunctions in the perturbed basis, respectively. Expanding the perturbed Hamiltonian and making the approximation that $\tilde{K}_{\tilde{v}\tilde{c},\tilde{v}'\tilde{c}'} \simeq K_{\tilde{v}\tilde{c},\tilde{v}'\tilde{c}'}$, the term in the bracket reads,

$$\sum_{\tilde{v}\tilde{c},\tilde{v}'\tilde{c}'} \langle vc|\tilde{v}\tilde{c}\rangle \langle \tilde{v}\tilde{c} \left| \tilde{H} \right| \tilde{v}'\tilde{c}'\rangle \langle \tilde{v}'\tilde{c}'|v'c'\rangle = \sum_{\tilde{v}\tilde{c}} \langle vc|\tilde{v}\tilde{c}\rangle (\epsilon_{\tilde{c}} - \epsilon_{\tilde{v}}) \langle \tilde{v}\tilde{c}|v'c'\rangle + K_{vc,v'c'}.$$

Next, we expand the perturbed wavefunction to the first order of the perturbation,

$$\left|\tilde{\phi}_i\right\rangle = |\phi_i\rangle + \delta\,|\phi\rangle = |\phi_i\rangle + \sum_{i \neq j} \frac{\langle i \left| \Delta V^i \right| j\rangle}{\epsilon_i - \epsilon_j} |\phi_j\rangle \equiv |\phi_i\rangle + \sum_{i \neq j} \Delta_{ij} |\phi_j\rangle .$$

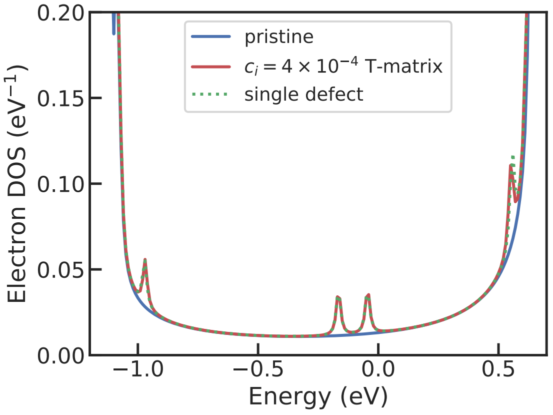

FIG. S3. Electronic density of states of the pristine monolayer MoS_2 (blue solid line) and that computed from diagonalizing the single-defect Hamiltonian (Green dots) and from the T-matrix approximation (red solid line). $c_i = 4 \times 10^{-4}$ ($N_i = 1$ defect per 48×48 unit cell) corresponds to a defect density of 5×10^{11} cm^{-2} for monolayer MoS_2.

We further take Tamm-Dancoff approximation and keep only the resonant part of the BSE Hamitonian so that the intersector terms $\Delta_{cv} = \Delta_{vc} = 0$. We obtain,

$$\langle vc|\tilde{v}\tilde{c}\rangle = \langle v|\tilde{v}\rangle\langle c|\tilde{c}\rangle = \left(\delta_{v\tilde{v}} + \sum_{v''\neq\tilde{v}} \Delta_{\tilde{v}v''}\delta_{v''v}\right)\left(\delta_{c\tilde{c}} + \sum_{c''\neq\tilde{c}} \Delta_{\tilde{c}c''}\delta_{c''c}\right) + \mathcal{O}(\Delta^2).$$

The $\langle\tilde{v}\tilde{c}|v'c'\rangle$ term is expanded similarly and we get the kinetic energy part of the perturbed Hamiltonian,

$$\sum_{\tilde{v}\tilde{c}} \langle vc|\tilde{v}\tilde{c}\rangle(\epsilon_{\tilde{c}} - \epsilon_{\tilde{v}})\langle\tilde{v}\tilde{c}|v'c'\rangle = (\epsilon_c - \epsilon_v)\,\delta_{vv'}\delta_{cc'} + \delta_{cc'}\,(\epsilon_{v'} - \epsilon_v)\,\Delta_{vv'} + \delta_{vv'}(\epsilon_c - \epsilon_{c'})\Delta^*_{cc'}.$$

Inserting equations above to the Eq. 1 and we obtain the perturbed BSE Hamiltonian in the exciton basis,

$$\tilde{H}_{mn} = \sum_{vc,v'c'} A^{S_m*}_{vc} A^{S_n}_{v'c'} \times \left[(\epsilon_c - \epsilon_v)\,\delta_{vv'}\delta_{cc'} + K_{vc,v'c'} + \delta_{cc'}\,(\epsilon_{v'} - \epsilon_v)\,\Delta_{vv'} + \delta_{vv'}(\epsilon_c - \epsilon_{c'})\Delta^*_{cc'}\right].$$

The first two terms in the bracket is the unperturbed Hamiltonian for excitons while the second term can be identified as the exciton-defect interaction

$$\begin{aligned} H_{ex-i} &= \sum_{vc,v'c'} A^{S_m*}_{vc} A^{S_n}_{v'c'}\left[\delta_{cc'}\,(\epsilon_{v'} - \epsilon_v)\,\Delta_{vv'} + \delta_{vv'}(\epsilon_c - \epsilon_{c'})\Delta^*_{cc'}\right] \\ &= \sum_{vc,v'c'} A^{S_m*}_{vc} A^{S_n}_{v'c'}\left[-\delta_{cc'}\left\langle v'\left|\Delta V^i\right| v\right\rangle + \delta_{vv'}\left\langle c\left|\Delta V^i\right| c'\right\rangle\right]. \end{aligned}$$

Restoring the momentum indices of Bloch states, we have

$$H_{ex-i} = \sum_{vc,v'c'} A^{S_m*\mathbf{k}_c-\mathbf{k}_v}_{v\mathbf{k}_v c\mathbf{k}_c} A^{S_n\mathbf{k}_{c'}-\mathbf{k}_{v'}}_{v'\mathbf{k}_{v'}c'\mathbf{k}_{c'}}\left[-\delta_{cc'}\left\langle v'\mathbf{k}_{v'}\left|\Delta V^i(\mathbf{q})\right| v\mathbf{k}_v\right\rangle + \delta_{vv'}\left\langle c\mathbf{k}_c\left|\Delta V^i(\mathbf{q})\right| c'\mathbf{k}_{c'}\right\rangle\right].$$

Momentum conservation imposes the constraints $\delta(\mathbf{k}_{c'} + \mathbf{q} - \mathbf{k}_c)$ for the second term and $\delta(\mathbf{k}_v + \mathbf{q} - \mathbf{k}_{v'})$ for the first term. We define the electron-defect matrix elements in the Bloch basis

$$V_{ij}(\mathbf{k},\mathbf{q}) = \left\langle j\mathbf{k}+\mathbf{q}\left|\Delta V^i(\mathbf{q})\right| i\mathbf{k}\right\rangle.$$

The exciton-defect Hamiltonian then reads

$$\sum_{vc,v'c'} A^{S_m*\mathbf{k}_c-\mathbf{k}_v}_{v\mathbf{k}_v c\mathbf{k}_c} A^{S_n\mathbf{k}_{c'}-\mathbf{k}_{v'}}_{v'\mathbf{k}_{v'}c'\mathbf{k}_{c'}} \left[-\delta_{cc'}\left\langle v'\mathbf{k}_{v'}\left|\Delta V^i(\mathbf{q})\right|v\mathbf{k}_v\right\rangle + \delta_{vv'}\left\langle c\mathbf{k}_c\left|\Delta V^i(\mathbf{q})\right|c'\mathbf{k}_{c'}\right\rangle\right]$$
$$= \sum_{\mathbf{k}\mathbf{k}_c\mathbf{k}_{c'}\mathbf{q}} \sum_{vcc'} A^{S_m*\mathbf{k}_c-\mathbf{k}}_{v\mathbf{k}c\mathbf{k}_c} A^{S_n\mathbf{k}_{c'}-\mathbf{k}}_{v\mathbf{k}c'\mathbf{k}_{c'}} \left\langle c\mathbf{k}_c\left|\Delta V^i(\mathbf{q})\right|c'\mathbf{k}_{c'}\right\rangle$$
$$- \sum_{\mathbf{k}\mathbf{k}_c\mathbf{k}_{c'}} \sum_{vc,v'c'} A^{S_m*\mathbf{k}_c-\mathbf{k}_v}_{v\mathbf{k}_v c\mathbf{k}_c} A^{S_n\mathbf{k}_{c'}-\mathbf{k}_{v'}}_{v'\mathbf{k}_{v'}c'\mathbf{k}_{c'}} \delta_{cc'}\left\langle v'\mathbf{k}_{v'}\left|\Delta V^i(\mathbf{q})\right|v\mathbf{k}_v\right\rangle$$
$$= \sum_{\mathbf{k}\mathbf{Q}\mathbf{q}}\left[\sum_{vcc'} A^{S_m*\mathbf{Q}+\mathbf{q}}_{v\mathbf{k}c\mathbf{k}+\mathbf{Q}+\mathbf{q}} A^{S_n\mathbf{Q}}_{v\mathbf{k}c'\mathbf{k}+\mathbf{Q}} V_{c'c}(\mathbf{k}+\mathbf{Q},\mathbf{q}) - \sum_{vcv'} A^{S_m*\mathbf{Q}+\mathbf{q}}_{v\mathbf{k}-\mathbf{q}c\mathbf{k}+\mathbf{Q}} A^{S_n\mathbf{Q}}_{v'\mathbf{k}c'\mathbf{k}+\mathbf{Q}} V_{vv'}(\mathbf{k}-\mathbf{q},\mathbf{q})\right]$$

where we set $\mathbf{k}_v = \mathbf{k}$, $\mathbf{k}_{c'} = \mathbf{k}+\mathbf{Q}$, and impose the constraint $\mathbf{k}_c = \mathbf{k}_{c'}+\mathbf{q}$ in the first sum and $\mathbf{k}_{v'} = \mathbf{k}$, $\mathbf{k}_{c'} = \mathbf{k}+\mathbf{Q}$, $\mathbf{k}_{v'} = \mathbf{k}_v+\mathbf{q}$ in the second sum. The exciton-defect interaction matrix elements can then be defined as,

$$V_{m\mathbf{Q}+\mathbf{q},n\mathbf{Q}} = \sum_{\mathbf{k}}\left[\sum_{vcc'} A^{S_m*\mathbf{Q}+\mathbf{q}}_{v\mathbf{k}c\mathbf{k}+\mathbf{Q}+\mathbf{q}} A^{S_n\mathbf{Q}}_{v\mathbf{k}c'\mathbf{k}+\mathbf{Q}} V_{c'c}(\mathbf{k}+\mathbf{Q},\mathbf{q}) - \sum_{vcv'} A^{S_m*\mathbf{Q}+\mathbf{q}}_{v\mathbf{k}-\mathbf{q}c\mathbf{k}+\mathbf{Q}} A^{S_n\mathbf{Q}}_{v'\mathbf{k}c'\mathbf{k}+\mathbf{Q}} V_{vv'}(\mathbf{k}-\mathbf{q},\mathbf{q})\right].$$

The exiton-defect interaction Hamiltonian reads,

$$H_{ex-d} = \sum_{\mathbf{Q}\mathbf{q}nm} V_{nm}(\mathbf{Q},\mathbf{q}) a^\dagger_{m\mathbf{Q}+\mathbf{q}} a_{n\mathbf{Q}},$$

which appears in the Eq. 1 in the main text.

Before closing this section, we note that a different scheme to compute exciton-defect interaction self-energy without writing down the explicit interaction Hamiltonian is tempting. One could first compute defected-averaged electron propagators then solve the BSE constructed on top of those. However, based on the diagrammatic analysis in Ref. [13] and Ref. [14] in the context of exciton-phonon interactions, diagrams with exciton-defect scattering lines connecting electron and hole propagators would be neglected, which include the diagrams with excitonic effects in the intermediate states.

DEFECT DENSITY DEPENDENCE OF SELF-ENERGY AND GREEN FUNCTION

In Fig. 2 in the main text we show the Born and T-matrix selfenergy and the real part of the Green functions of A exciton at two defect densities of 5×10^{11} cm^{-2} and 2.5×10^{12} cm^{-2}, which corresponds to $N_i = 1$ and $N_i = 5$ defect per supercell, respectively. Here, we show the comparison of the selfenergy and the Green function on a wider range of defect density in Fig. S4 and Fig. S5.

Both selfenergies are proportional to the defect density as shown in Eq. 3 and 4 in the main text after defect-averaging. The Born selfenergy mainly has intensity near the bare A exciton energy and shows a peak at that energy. As the defect density increases, the renormalized A exciton energy shifts to the lower energy side. In contrast, a few peaks develop away from the bare A exciton energy in the T-matrix selfenergy as increasing defect density. These peaks correspond to the defect-bound state as shown in Eq. S3 and discussed in Ref. [10] for electron-defect problems. At high defect density $N_i = 5$, we find a secondary peak around 1.5 eV and the splitting of A exciton peak. The energy renormalization can barely be seen compared with the Born selfenergy.

In the inset of Fig. S5 (d), we show the dependence of the peak of the imaginary part of the T-matrix selfenergy and the Bd1 peak on the defect density. In the low density limit the Bd1 peak locates exactly at the pole of the T-matrix.

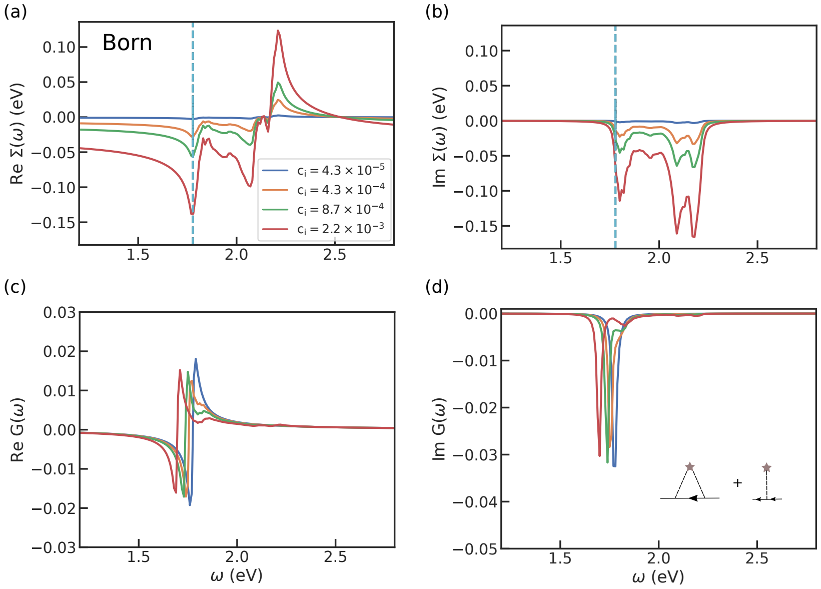

FIG. S4. (a) the real and (b) imaginary part of retarded Born self-energy of A exciton at various densities. (c) and (d) show the real and imaginary part of the Green's function, respectively. The blue dashed line indicates the bare A exciton energy. The defect densities are given as the number of defect per unit cell. The blue, orange, green, and red line correspond to results with numbers of defect $N_i = 0.1$, 1, 2, and 5 in a 48×48 supercell, respectively. Inset in (d) shows the self-energy diagrams in the approximation.

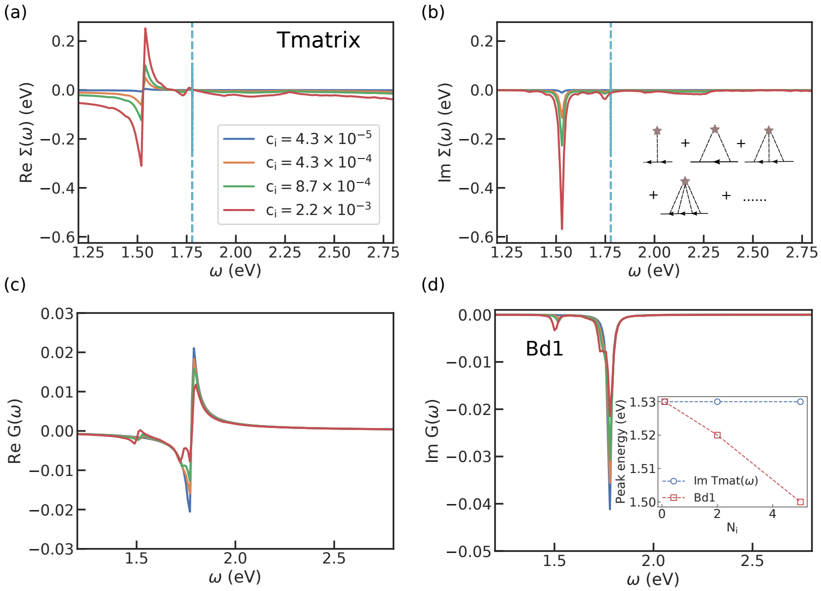

FIG. S5. Same as Fig. S4 but for T-matrix selfenergy. Inset in (b) shows the self-energy diagrams. Inset in (d) shows the dependence of the imaginary part of the peak of the T-matrix self-energy and the Bd1 peak on the number of defects N_i.

OPTICAL ABSORPTION SPECTRUM

The absorption spectrum can be simulated by the imaginary part of the dielectric function $\epsilon_2(\omega)$. A general expression for $\epsilon_2(\omega)$ is

$$\epsilon_2(\omega) = -\frac{e^2}{V_{tot}\epsilon_0}\mathrm{Im}\left[\sum_{ss'}\Omega_s^* G_{ss'}^R(\omega)\Omega_{s'}\right],$$

where $G^R(\omega)$ is the retarded part of the exciton Green's function. In the formulation of many-body perturbation theory, we can write the effective single exciton Hamiltonian as $H_{ss'}(\omega) = E_{s\mathbf{0}}\delta_{ss'} + \Sigma_{ss'}(\omega)$, where s and s' label bare exciton states, and the retarded component of the Green's function can be obtained by inverting the effective Hamiltonian

$$G_{ss'}^R(\omega) = \left[\frac{1}{\omega - H(\omega) + i\eta}\right]_{ss'}.$$

We solve the full Green's function by diagonalizing the effective Hamiltonian matrix and write

$$\tilde{H}_{\lambda\lambda'} = T_{\lambda s}^{-1} H_{ss'} T_{s'\lambda},$$

where $T_{s\lambda}$ is λ-th eigenvector and the eigenvalues $\tilde{H}_{\lambda\lambda'} = \left(\tilde{E}_\lambda + i\tilde{\Gamma}_\lambda\right)\delta_{\lambda\lambda'}$ can be complex. In terms of the eigenvectors of the effective Hamiltonian, the $\epsilon_2(\omega)$ reads,

$$\epsilon_2(\omega) = -\frac{e^2}{V_{tot}\epsilon_0}\mathrm{Im}\left[\sum_{\lambda ss'}\frac{\Omega_s^* T_{\lambda s}(\omega) T_{\lambda s'}^{-1}(\omega)\Omega_{s'}}{\hbar\omega - \tilde{E}_\lambda(\omega) - i\tilde{\Gamma}_\lambda(\omega)}\right].$$

LOCAL DENSITY OF STATES

In the main text we computed the real space electron-hole distribution of excitons given by

$$\rho(r_e, r_h, \omega) = -\frac{1}{\pi}\sum_S \mathrm{Im}G_{SS}^R(\omega)\left|\phi^S(r_e, r_h)\right|^2, \tag{S2}$$

where ϕ^S is the exciton wave function. In Fig. 3 (d) in the main text, we show the distribution with fixed hole position at the energy of the Bd1 peak in Fig. 3 (a) for a defect density of $2.5 \times 10^{12}\ \mathrm{cm}^{-2}$. We find that the electron distribution is similar to that of the A exciton despite the local charge density being a few times smaller, which is consistent with the state-decomposed spectral function shown in Fig. 3 (c). We note that since the defect-averaged T-matrix self-energy and Green's function both preserve translational symmetry, there is no preferential unit cell for the defect-bound exciton to localize.

PHOTOLUMINESCENCE

In contrast to the absorption spectrum, the photo-luminescence spectrum can be computed from the lesser component of the Green's function [15]

$$I_{PL}(\omega) = i\left(\frac{n_b\omega^3}{V\hbar\pi c^3}\right)\sum_s |\Omega_s|^2 G_{ss}^<(\omega),$$

where n_b is the refraction index. From the Kubo-Martin-Schwinger relation [16],

$$G_{sj}^<(\omega) = b(\omega)\left(G_{sj}^R(\omega) - G_{sj}^A(\omega)\right) = 2b(\omega)\mathrm{Im}G_{sj}^R(\omega),$$

and

$$\epsilon_2(\omega) \propto \mathrm{Im}\sum_s |\Omega_s|^2 G^R_{ss}(\omega).$$

we can connect the PL intensity spectrum to the absorption spectrum,

$$I_{PL}(\omega) \propto \omega^3 \epsilon_2(\omega) b(\omega),$$

where $b(\omega)$ is the Bose-Einstein distribution function. We found, however, that the expression above is difficult to evaluate numerically when $G^R(\omega)$ does not have a simple quasi-exciton structure and the full frequency dependence is nontrivial, e.g., in the case with satellites. To solve $G^<(\omega)$ numerically and avoid explicit evaluation of Bose functions, we derive an alternative expression for $G^<(\omega)$ in the next section.

Lesser Green's function and quasi-particle expansion

We start from $G^< = G^R T^< G^A$ [16] to derive an expression for $G^<$ for the exciton-defect problem. The retarded part of the T-matrix self-energy can be solved from the Dyson equation,

$$T^R = V + V\frac{1}{\omega - H_0 - V}V,$$

where we hide the frequency argument and exction states indices for clarity and write the bare Green's function in terms of the bare exciton Hamiltonian, H_0. The matrix elements of self-energy can be expanded by the eigenvectors U of the full exciton-defect Hamiltonian $H = H_0 + V$, solved by $\sum_{S\mathbf{Q}'} H_{S_n\mathbf{Q}S_m\mathbf{Q}'}U^\lambda_{S_m\mathbf{Q}'} = E^\lambda U^\lambda_{S_n\mathbf{Q}}$. We write

$$\begin{aligned} T^R_{S_n\mathbf{Q}S_m\mathbf{Q}'} &= V_{S_n\mathbf{Q}S_m\mathbf{Q}'} + \sum_\lambda \left[\frac{1}{\omega - E^\lambda + i\eta}\left(\sum_{S_l\mathbf{Q}''} V_{S_n\mathbf{Q}S_l\mathbf{Q}''}U^\lambda_{S_l\mathbf{Q}''}\right)\left(\sum_{S_o\mathbf{Q}'''}(U^\dagger)^\lambda_{S_o\mathbf{Q}'''}V_{S_o\mathbf{Q}'''S_m\mathbf{Q}'}\right)\right] \\ &= V_{S_n\mathbf{Q}S_m\mathbf{Q}'} + \sum_\lambda \frac{W^\lambda_{S_n\mathbf{Q}}W^{\lambda,\dagger}_{S_m\mathbf{Q}'}}{\omega - E^\lambda + i\eta}, \end{aligned} \tag{S3}$$

where we define $W^\lambda_{S_n\mathbf{Q}} = \sum_{S_l\mathbf{Q}''} V_{S_n\mathbf{Q}S_l\mathbf{Q}''}U^\lambda_{S_l\mathbf{Q}''}$. We have confirmed from numerics that the solution of Eq. S3 is identical to that of the Dyson's equation, Eq. 4 in the main text.

The lesser part of the self-energy can be solved from the KMS relation,

$$\begin{aligned} T^<_{S_n\mathbf{Q}S_m\mathbf{Q}'}(\omega) &= 2ib(\omega)\mathrm{Im}T^R_{S_n\mathbf{Q}S_m\mathbf{Q}'}(\omega) \\ &= -2\pi i b(\omega)\sum_\lambda W^\lambda_{S_n\mathbf{Q}}W^{\lambda,\dagger}_{S_m\mathbf{Q}'}\delta(\omega - E^\lambda), \end{aligned}$$

from which, we use $G^<_{S\mathbf{Q}}(\omega) = G^R_{S\mathbf{Q}}(\omega)T^<_{S\mathbf{Q}}(\omega)G^A_{S\mathbf{Q}}(\omega)$ to solve for the lesser Green's function

$$\begin{aligned} G^<_{S_nS_m\mathbf{Q}}(\omega) = &-2\pi i\left(\frac{1}{\omega - H_0 - \Sigma^R(\omega) + i\eta}\right)_{S_nS_l\mathbf{Q}}\left(\frac{1}{\omega - H_0 - \Sigma^A(\omega) - i\eta}\right)_{S_aS_m\mathbf{Q}} \\ &\times \sum_\lambda W^\lambda_{S_l\mathbf{Q}}W^{\lambda,\dagger}_{S_a\mathbf{Q}}\delta(\omega - E^\lambda)b(\omega). \end{aligned} \tag{S4}$$

Although it is possible to evaluate Eq. S4 numerically, its connection to bare exciton states is not recognizable. To make the pole structure apparent, we adopt the quasi-particle (QP) expansion of $G^<$ following Ref. [17, 18], which separates the QP contribution from the dynamical one. It can be shown that

$$G^<_{S\mathbf{Q}}(\omega) = G^{QP,<}_{S\mathbf{Q}}(\omega)(1 - R_{S\mathbf{Q}}) + G^{QP,R}_{S\mathbf{Q}}(\omega)\Sigma^<_{S\mathbf{Q}}(\omega)G^{QP,A}_{S\mathbf{Q}}(\omega),$$

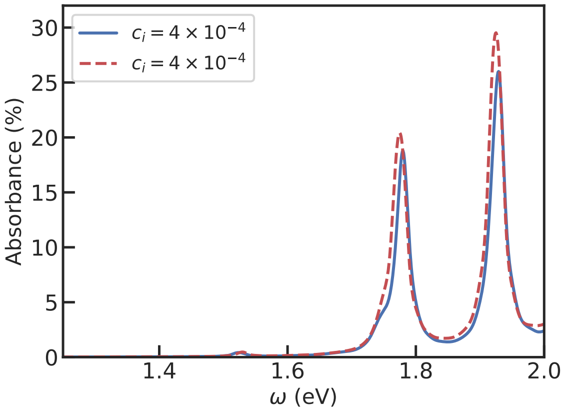

FIG. S6. Absorbance of monolayer MoS_2 with a defect density of $c_i = 4\times10^{-4}$ per unit cell ($N_i = 1$ per 48×48 unit cells or 5×10^{11} cm^{-2}). computed from the full retarded Green's function (blue solid line) and from QP expansion (red dashed line).

where $R_{S\mathbf{Q}} = -\partial_\omega \mathrm{Re}\Sigma_{S\mathbf{Q}}(\omega)|_{\omega=E^{QP}_{S\mathbf{Q}}}$ is the renormalization factor and $E^{QP}_{S\mathbf{Q}} = E_{S\mathbf{Q}} + \mathrm{Re}\Sigma^R_{S\mathbf{Q}}(E^{QP}_{S\mathbf{Q}})$. In practice, $G^{QP}_{S\mathbf{Q}}$ is obtained replacing $E_{S\mathbf{Q}}$ with $E^{QP}_{S\mathbf{Q}}$ in the bare Green's function so that

$$G^{QP,<}_{S\mathbf{Q}}(\omega) = -2\pi i b(\omega)\delta(\omega - E^{QP}_{S\mathbf{Q}})$$

and

$$G^{QP,R/A}_{S\mathbf{Q}}(\omega) = \frac{1}{\omega - E^{QP}_{S\mathbf{Q}} \pm i\eta}.$$

Finally, inserting $G^{QP,<}$, $G^{QP,R/A}$, and $T^<$ into Eq. $G^<$, we obtain

$$\begin{aligned} G^<_{S\mathbf{Q}}(\omega) &= -2\pi i b(\omega)\delta(\omega - E^{QP}_{S\mathbf{Q}})(1 - 2R_{S\mathbf{Q}}) \\ &- 2\pi i \frac{N_i}{(\omega - E^{QP}_{S\mathbf{Q}})^2 + \eta^2} \sum_\lambda b(\omega) \left|W^\lambda_{S\mathbf{Q}}\right|^2 \delta(\omega - E^\lambda), \end{aligned} \tag{S5}$$

where N_i is the number of impurtiy and we have assumed both Green's functions and the self-energy are diagonal in the exciton state index. Eq. S5 now clearly shows the renormalized quasi-exciton peaks in the first term and the defect-induced dynamical structure in the second term. PL spectra in Fig. 4 in the main text are computed with Eq. S5 by approximating delta functions with Lorentzian functions of a 10 meV broadening.

QP expansion of the imaginary part of the retarded Green's function can be obtained from KMS relation. We have

$$\begin{aligned} \mathrm{Im}G^R_{S\mathbf{Q}}(\omega) &= -\pi\delta(\omega - E^{QP}_{S\mathbf{Q}})(1 - 2R_{S\mathbf{Q}}) \\ &- \pi \frac{N_i}{(\omega - E^{QP}_{S\mathbf{Q}})^2 + \eta^2} \sum_\lambda \left|W^\lambda_{S\mathbf{Q}}\right|^2 \delta(\omega - E^\lambda). \end{aligned}$$

In Fig. S6, we compare the absorbance spectra computed from full G^R solution and from the QP expansion. We find that the QP expansion is a good approximation already at $N_i = 1$, which justifies the expansion we used for the lesser Green's function.

Kubo-Martin-Schwinger relation

We check the KMS relation for the one-shot T-matrix calculation. The Kadanoff-Baym (KB) equation for the lesser and the retarded component of the T-matrix reads [16]

$$T^<(\omega) = VG^<_0(\omega)T^A(\omega) + VG^R_0(\omega)T^<(\omega).$$

and

$$T^{R/A}(\omega) = V + VG_0^{R/A}(\omega)T^{R/A}(\omega),$$

respectively. We start from the KMS relation for the bare Green's function

$$G_0^<(\omega) = b(\omega)(G_0^R - G_0^A).$$

Inserting the KMS equation for $G_0^<$ into the KB equation for $T^<$, we have

$$\begin{aligned} T^< &= Vb(\omega)G_0^R T^A - Vb(\omega)G_0^A T^A + VG_0^R T^< \\ &= VG_0^R(b(\omega)T^A + T^<) - Vb(\omega)G_0^A T^A. \end{aligned}$$

We can show that KMS relation is consistent with the equation above by using the KMS relation $T^<$ on the right-hand side of the equation

$$\begin{aligned} T^< &= VG_0^R(b(\omega)T^A + T^<) - Vb(\omega)G_0^A T^A \\ &= VG_0^R b(\omega)T^R - Vb(\omega)G_0^A T^A \\ &= b(\omega)(T^R - T^A). \end{aligned}$$

Alternatively, from the KB equation,

$$T^<(1 - G_0^A V) = T^R G_0^< V,$$

we have

$$\begin{aligned} T^< &= T^R G_0^< V (G_0^A)^{-1} \frac{1}{(G_0^A)^{-1} - V} \\ &= T^R G_0^< V + T^R G_0^< V \frac{1}{(G_0^A)^{-1} - V} V \\ &= T^R G_0^< \left(V + V \frac{1}{(G_0^A)^{-1} - V} V \right) \\ &= T^R G_0^< T^A. \end{aligned}$$

By inverting Dyson's equation

$$T^A = V + VG_0^A T^A \qquad T^R = V + T^R G_0^R V,$$

we have

$$G_0^A T^A = V^{-1} T^A - 1 \qquad T^R G_0^R = T^R V^{-1} - 1.$$

Using these equations, we prove the KMS relation for $T^<$

$$\begin{aligned} T^< &= T^R G_0^< T^A \\ &= T^R b(\omega)(G_0^R - G_0^A)T^A \\ &= b(\omega)(T^R G_0^R T^A - T^R G_0^A T^A) \\ &= b(\omega)\left(T^R V^{-1} T^A - T^A - T^R V^{-1} T^A + T^R\right) \\ &= b(\omega)(T^R - T^A). \end{aligned}$$

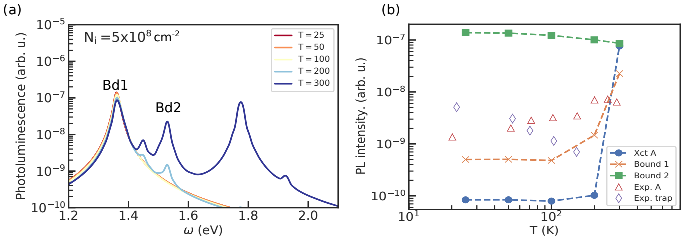

FIG. S7. Simulated photoluminescence spectra at (a) at different temperature with a fixed defect density of $N_i = 5 \times 10^8$ cm^{-2}. (b) Temperature dependences of the PL intensity at A exciton peak (blue dots), Bd1 (orange crosses), and Bd2 (green squares) peaks. Experimental data in (b) is adopted from Ref. [19]. The experimental data in (b) is scaled by an overall factor.

TEMPERATURE DEPENDENCE OF THE PL SPECTRUM

In Fig. S7 (a) we study the temperature dependence of the PL intensity for a low defect density of 5×10^{-8} cm^{-2}. We find that the PL emission is completely governed by Bd1 state at low temperature due to the large population of excitons. A exction and Bd2 state emissions become comparable to the Bd1 peak only when the temperature reaches 300 K while the intensity of Bd1 state gradually decreases with increasing temperature. The temperature dependences of A, Bd1, and Bd2 peak intensity are shown in Fig. S7 (b). Compared with the experiments from Ref. [19], although the general trend agrees, the experimental data shows a weaker temperature dependence. The discrepancy may be due to the larger defect binding energy from our calculations, which results in a spectrum dominated by low-energy defect bound states within a wider range of temperature. Moreover, temperature effects appear only through the Bose factor in Eq. S5, where the energy shift and linewidth changes due to exciton-phonon couplings observed in the experiment are completely ignored.

* yanghao@gate.sinica.edu.tw
† diana.qiu@yale.edu
‡ jornada@stanford.edu

[1] Paolo Giannozzi, Stefano Baroni, Nicola Bonini, Matteo Calandra, Roberto Car, Carlo Cavazzoni, Davide Ceresoli, Guido L Chiarotti, Matteo Cococcioni, Ismaila Dabo, Andrea Dal Corso, Stefano de Gironcoli, Stefano Fabris, Guido Fratesi, Ralph Gebauer, Uwe Gerstmann, Christos Gougoussis, Anton Kokalj, Michele Lazzeri, Layla Martin-Samos, Nicola Marzari, Francesco Mauri, Riccardo Mazzarello, Stefano Paolini, Alfredo Pasquarello, Lorenzo Paulatto, Carlo Sbraccia, Sandro Scandolo, Gabriele Sclauzero, Ari P Seitsonen, Alexander Smogunov, Paolo Umari, and Renata M Wentzcovitch, "Quantum espresso: a modular and open-source software project for quantum simulations of materials," Journal of Physics: Condensed Matter **21**, 395502 (2009).

[2] Jin-Jian Zhou, Jinsoo Park, I-Te Lu, Ivan Maliyov, Xiao Tong, and Marco Bernardi, "Perturbo: A software package for ab initio electron–phonon interactions, charge transport and ultrafast dynamics," Computer Physics Communications **264**, 107970 (2021).

[3] I-Te Lu, Jin-Jian Zhou, and Marco Bernardi, "Efficient ab initio calculations of electron-defect scattering and defect-limited carrier mobility," Phys. Rev. Mater. **3**, 033804 (2019).

[4] I.-Te Lu, Jinsoo Park, Jin-Jian Zhou, and Marco Bernardi, "Ab initio electron-defect interactions using wannier functions," npj Computational Materials **6**, 17 (2020).

[5] Jack Deslippe, Georgy Samsonidze, David A. Strubbe, Manish Jain, Marvin L. Cohen, and Steven G. Louie, "Berkeleygw: A massively parallel computer package for the calculation of the quasiparticle and optical properties of materials and nanostructures," Computer Physics Communications **183**, 1269–1289 (2012).

[6] Mark S. Hybertsen and Steven G. Louie, "Electron correlation in semiconductors and insulators: Band gaps and quasiparticle energies," Phys. Rev. B **34**, 5390–5413 (1986).

[7] Michael Rohlfing and Steven G. Louie, "Electron-hole excitations and optical spectra from first principles," Phys. Rev. B **62**, 4927–4944 (2000).

[8] Diana Y. Qiu, Ting Cao, and Steven G. Louie, "Nonanalyticity, valley quantum phases, and lightlike exciton dispersion in monolayer transition metal dichalcogenides: Theory and first-principles calculations," Phys. Rev. Lett. **115**, 176801 (2015).

[9] Yang-hao Chan, Jonah B. Haber, Mit H. Naik, Jeffrey B. Neaton, Diana Y. Qiu, Felipe H. da Jornada, and Steven G. Louie, "Exciton lifetime and optical line width profile via exciton-phonon interactions: Theory and first-principles calculations for monolayer mos2," Nano Lett. **23**, 3971–3977 (2023).

[10] Kristen Kaasbjerg, "Atomistic t-matrix theory of disordered two-dimensional materials: Bound states, spectral properties, quasiparticle scattering, and transport," Phys. Rev. B **101**, 045433 (2020).

[11] Junqing Xu, Adela Habib, Ravishankar Sundararaman, and Yuan Ping, "Ab initio ultrafast spin dynamics in solids," Phys. Rev. B **104**, 184418 (2021).

[12] Hsiao-Yi Chen, Davide Sangalli, and Marco Bernardi, "Exciton-phonon interaction and relaxation times from first principles," Phys. Rev. Lett. **125**, 107401 (2020).

[13] Gabriel Antonius and Steven G. Louie, "Theory of exciton-phonon coupling," Phys. Rev. B **105**, 085111 (2022).

[14] Pierluigi Cudazzo, "First-principles description of the exciton-phonon interaction: A cumulant approach," Phys. Rev. B **102**, 045136 (2020).

[15] K. Hannewald, S. Glutsch, and F. Bechstedt, "Theory of photoluminescence in semiconductors," Phys. Rev. B **62**, 4519–4525 (2000).

[16] Gianluca Stefanucci and Robert van Leeuwen, *Nonequilibrium Many-Body Theory of Quantum Systems: A Modern Introduction* (Cambridge University Press, 2013).

[17] F. Bechstedt, M. Fiedler, C. Kress, and R. Del Sole, "Dynamical screening and quasiparticle spectral functions for nonmetals," Phys. Rev. B **49**, 7357–7362 (1994).

[18] Pierluigi Cudazzo, "Dynamical effects on photoluminescence spectra from first principles: A many-body green's function approach," Phys. Rev. B **108**, 165101 (2023).

[19] Ke Wu, Hongxia Zhong, Quanbing Guo, Jibo Tang, Zhenyu Yang, Lihua Qian, Shengjun Yuan, Shunping Zhang, and Hongxing Xu, "Revealing the competition between defect-trapped exciton and band-edge exciton photoluminescence in monolayer hexagonal ws2," Advanced Optical Materials **10**, 2101971 (2022), https://advanced.onlinelibrary.wiley.com/doi/pdf/10.1002/adom.202101971.
